# Regulation of brown adipocyte metabolism by myostatin/follistatin signaling

**DOI:** 10.3389/fcell.2014.00060

**Published:** 2014-10-16

**Authors:** Rajan Singh, Melissa Braga, Shehla Pervin

**Affiliations:** ^1^Division of Endocrinology and Metabolism, Charles R. Drew University of Medicine and ScienceLos Angeles, CA, USA; ^2^Department of Obstetrics and Gynecology, David Geffen School of Medicine at UCLALos Angeles, CA, USA

**Keywords:** brown adipose tissue, beige fat, obesity, myostatin, follistatin

## Abstract

Obesity develops from perturbations of cellular bioenergetics, when energy uptake exceeds energy expenditure, and represents a major risk factor for the development of type 2 diabetes, dyslipidemia, cardiovascular disease, cancer, and other conditions. Brown adipose tissue (BAT) has long been known to dissipate energy as heat and contribute to energy expenditure, but its presence and physiological role in adult human physiology has been questioned for years. Recent demonstrations of metabolically active brown fat depots in adult humans have revolutionized current therapeutic approaches for obesity-related diseases. The balance between white adipose tissue (WAT) and BAT affects the systemic energy balance and is widely believed to be the key determinant in the development of obesity and related metabolic diseases. Members of the transforming growth factor-beta (TGF-β) superfamily play an important role in regulating overall energy homeostasis by modulation of brown adipocyte characteristics. Inactivation of TGF-β/Smad3/myostatin (Mst) signaling promotes browning of white adipocytes, increases mitochondrial biogenesis and protects mice from diet-induced obesity, suggesting the need for development of a novel class of TGF-β/Mst antagonists for the treatment of obesity and related metabolic diseases. We recently described an important role of follistatin (Fst), a soluble glycoprotein that is known to bind and antagonize Mst actions, during brown fat differentiation and the regulation of cellular metabolism. Here we highlight various investigations performed using different *in vitro* and *in vivo* models to support the contention that targeting TGF-β/Mst signaling enhances brown adipocyte functions and regulates energy balance, reducing insulin resistance, and curbing the development of obesity and diabetes.

## Introduction

Obesity is a major health problem spreading at an epidemic pace throughout the world without any sign of abatement. According to the World Health Organization report, more than 1 billion adults (~ 15% of world population) are overweight [body mass index (BMI) > 25] (Tseng et al., 2010). Over 300 million adults are essentially obese (BMI > 30), and these numbers are expected to increase by more than 50% by the year 2015 (Tseng et al., 2010). Understanding the cellular process responsible for metabolic perturbations that contribute to the expansion of adipose tissues accompanying obesity is critical for the development of therapeutic interventions. Any treatment for obesity must reduce total energy uptake, increase energy expenditure, or have an effect on both of these processes. Standard clinical approaches such as calorie restriction and exercise programs have limited effectiveness. Anti-obesity drugs targeted against the site of excess fat storage are mainly viewed as weight-loss drugs that modify the central nervous system (Grundy, 2006). The potential strategies to achieve weight-loss are to reduce energy intake by stimulating anorexigenic signals or by blocking orexigenic signals and to increase energy expenditure (Isidro and Cordido, 2009). Although such drugs have been used as anti-obesity drugs, only modest weight loss for relatively shorter periods of time has been achieved (Grundy, 2006). Therapeutic strategies available to combat the increased rate of obesity are inadequate because of the inherent resistance of human body to weight loss (Whittle et al., 2011). At the same time, several safety concerns and issues related to associated toxicity have led to the withdrawal of some of these drugs (Ioannides-Demos et al., 2006; Elangbam, 2009). Targeting energy expenditure pathways by activating BAT is therefore conceived as a promising alternative strategy that could provide significant potential health benefits when used either alone or in combination with other approaches (Boss and Farmer, 2012; Townsend and Tseng, 2014). Accordingly, there is a growing interest in identifying such classes of drugs which would increase adaptive thermogenesis by increasing brown fat differentiation from progenitor cells, activate brown fat, and skeletal muscle thermogenesis, or increase general mitochondrial uncoupling (Wu et al., 2012; Harms and Seale, 2013).

## Adipose tissue browning and energy metabolism

Metabolically active depots of brown adipose tissue (BAT) have recently been identified in adult humans (Nedergaard et al., 2007; van Marken Lichtenbelt et al., 2009; Virtanen et al., 2009). BAT exists in distinct locations in small animals and is innervated by the sympathetic nervous system (SNS) (Farmer, 2008). In sharp contrast to white adipose tissue (WAT) which is known to store energy in the form of triglycerides in periods of excess energy intake, BAT metabolizes stored energy to generate heat for thermogenic purposes (Ouellet et al., 2012; Virtanen, 2014). The dynamic process of fat deposition and mobilization are therefore, two key functions of adipose tissues. Genetic fat-mapping experiments indicate that BAT in the interscapular region and skeletal muscle but not white adipose cells arise from Myf5^+^ precursor cells (Kajimura et al., 2010). On the other hand, “brown-like” fat cells are derived from non-Myf5 (Myf5^−^) lineage and are called beige cells or brite (brown-in-white) cells (Seale et al., 2008; Kajimura et al., 2010). However, this concept has recently been challenged. Studies based on lineage analysis suggest that subsets of white adipocytes originates from both Myf5^+^ and Myf5^−^ precursors and respond to β3-adrenoceptor stimulation and therefore, brite adipocytes may also have multiple origins (Sanchez-Gurmaches et al., 2012; Lee and Cowan, 2013; Rosenwald et al., 2013). These brite cells possess the morphological and biochemical characteristics of classical brown adipocytes, including the expression of UCP1 and multilocular lipid droplets (Ohno et al., 2012; Kajimura and Saito, 2014). Transdifferentiation allows efficient conversion of white adipocytes to brown adipocytes and vice versa (Frontini and Cinti, 2010; Yadav et al., 2011). In general, cold exposure conditions promote white-to-brown adipocyte conversion through beta (3)-adrenoceptor-mediated transdifferentiation (Bartelt et al., 2011). A recent report also demonstrated a bi-directional interconversion of brite and white adipocytes (Rosenwald et al., 2013). Brite adipocytes change their morphology and gene expression profile to that of white adipocytes under warm adaptation, or after high fat diet, a conversion that is not observed in classical brown adipocytes (Rosenwald et al., 2013). Prospective studies in humans found detectable BAT in up to 96% of healthy young adults but less often in older or obese subjects (Saito et al., 2009; van Marken Lichtenbelt et al., 2009; Ouellet et al., 2011). Human brown adipocytes possess molecular signatures that resemble brite cells (Sharp et al., 2012; Wu et al., 2012). It is, therefore, logical to speculate that human adipocytes might convert from brite into white adipocyte phenotype during aging. There is a growing list of compounds that induce browning of white adipocytes and therefore, have the ability to modulate overall energy metabolism (Nedergaard and Cannon, 2014). Therapeutic potential of both kinds of adipose cells which are regulated differently have been demonstrated. Genetic manipulations to induce more brown or beige fat are associated with significant anti-obesity and anti-diabetic actions in mice (Wu et al., 2012; Harms and Seale, 2013). Upon cold exposure, heat production in the animals is facilitated by the release of catecholamines, which activate thermogenesis and the dissipation of heat. This specialized function of BAT is derived from high mitochondrial content and the ability to express uncoupling protein-1 (UCP 1), a proton transporter that uncouples electron transport from ATP production, allowing the energy to dissipate as heat (Ohno et al., 2012; Wu et al., 2012). Transgenic mice expressing UCP1 from the fatty acid binding protein 4 (FABP4) promoter are resistant to genetic and diet-induced obesity (Kopecky et al., 1995). Several transcriptional factors and cofactors affect the expression of Ucp1, and other key brown fat-selective genes have been identified. PRDM16, a 140kD zinc-finger protein is highly enriched in BAT compared to WAT. This protein is also shown to be selectively expressed in subcutaneous white adipocytes relative to other white fat depots (Seale et al., 2011). Ectopic expression of PRDM16 induces a nearly complete brown fat genetic program, including mitochondrial biogenesis, increased cellular respiration, and expression of brown-selective genes (Seale et al., 2008). Differential gene expression analysis between beige and brown fat cells demonstrates a related but distinctly clear expression profile (Wu et al., 2012). Epithelial V-like antigen 1 (EVA1) was highly enriched in brown fat cells, while developmental transcription factor (Tbx1), immune and inflammatory response molecules such as Tbx1, and CD137, Tmem26, and Slc27a1 have been identified as key beige-selective markers (Wu et al., 2012). Increased inflammation promotes alternative activation of macrophages and supports the development of functional beige cells (Ganeshan and Chawla, 2014; Mauer et al., 2014; Qiu et al., 2014).

## TGF-β superfamily, brown fat, and obesity

### TGF-β Smad3/activin receptor IIB signaling and energy metabolism

The transforming growth factor-β (TGF-β) superfamily is comprised of over 30 members that include several structurally dimeric cytokines such as TGF-β, activins, and bone morphogenic proteins (BMPs)/growth and differentiation factors (GDFs) which are evolutionarily highly conserved (Shi and Massagué, 2003). TGF-β transmits its signals via dual serine/threonine kinase receptors and transcription factors called Smads. Several direct and indirect links between TGF-β and mitochondrial energy metabolism and energy sensing pathways have been reported (Casalena et al., 2012). TGF-β overexpression deregulates the cellular redox state by stimulating reactive oxygen species (ROS) production through Nox4 activation and compromises the antioxidant system that protect against mitochondrial derived ROS by inhibiting the expression of MnSOD (Michaeloudes et al., 2011). TGF-β also modulates energy metabolism by controlling mitochondrial metabolism directly by reduction of complex IV and mitochondrial respiration that results in increased ROS production (Yoon et al., 2005). Extensive interaction between TGF-β and key energy sensors adenosine monophosphate-activated protein kinase (AMPK) and sirtuin family members has been demonstrated (Mishra et al., 2008; Verdin et al., 2010). TGF-β levels correlate with obesity in mice (Samad et al., 1999) and humans (Alessi et al., 2000; Fain et al., 2005). Furthermore, TGF-β/Smad3 pathway has also been demonstrated in the regulation of insulin gene transcription and β-cell function (Lin et al., 2009). Most recently, a comprehensive study highlighted the critical involvement of TGF-β/Smad3 signaling during the process of obesity and demonstrated the beneficial effects of systemic blockade of this signaling from obesity, diabetes, and hepatic steatosis (Yadav et al., 2011). Using hyperinsulinemic-euglycemic clamp experiments, this group reported that Smad3 loss protects experimental mice from diet-induced obesity and insulin resistance. This effect was associated with the phenotypic transition of white to BATs, induction of mitochondrial biogenesis, and enhanced expression of BAT-related gene signatures. Furthermore, elevated plasma TGF-β1 levels were shown to correlate positively with BMI and increased adiposity in overweight and obese subjects compared to subjects with normal BMI. Inhibition of activin receptor IIB (ActRIIB) that integrates actions of TGF-β related ligands via dimerization with Alk4/5 and signals intracellularly via Smad2/3 promotes differentiation of primary brown adipocytes *in vitro* and increases the amount of brown but not white fat in mice (Fournier et al., 2012). This blockade of ActRIIB enhanced mitochondrial function and uncoupled respiration resulted in increased energy expenditure under ambient or cold temperatures but not at thermoneutrality. Interestingly, molecular signatures induced by inhibition of ActRIIB overlap with PGC-1α overexpression *in vivo*. PGC-1α was originally described as a co-activator of PPAR-γ that modulated expression of key brown fat specific UCP-1 and thermogenesis in brown fat (Boström et al., 2012).

### Myostatin regulation of brown fat and energy metabolism

**Figure 1:**
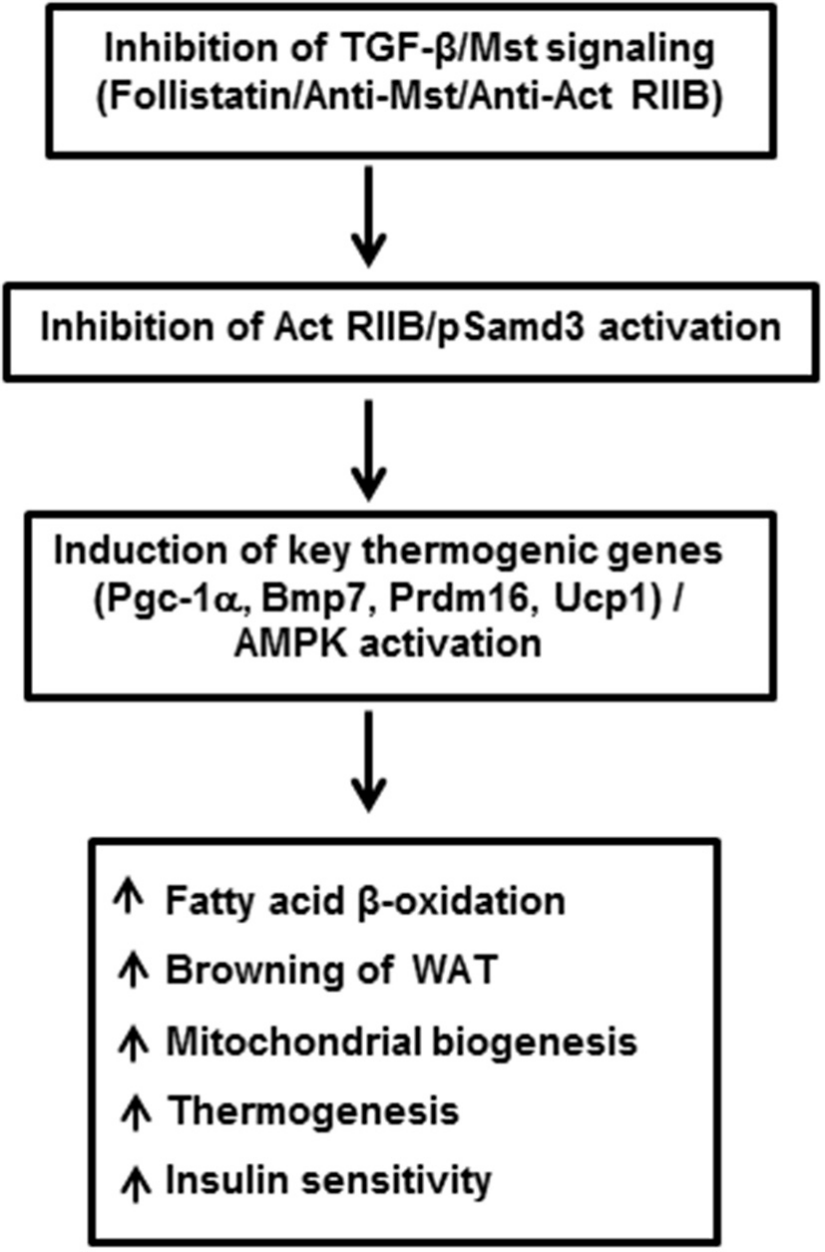
**Inhibition of TGF-β/Mst/Smad3 signaling induces browning of white adipocytes and regulates overall energy metabolism**. Act RIIB: Activin receptor type IIB; Pgc-1α: peroxisomal proliferator-activated receptor (PPAR) coactivator 1; Bmp7: bone morphogenic protein 7; Prdm16: PR domain containing 16; Ucp1: uncoupling protein 1; AMPK: adenosine monophosphate-activated protein kinase; WAT: white adipose tissue.

Myostatin (Mst), also termed as growth/differentiation factor-8 (GDF-8) is a member of the TGF-β superfamily that regulates skeletal muscle mass (McPherron et al., 1997). Mst-deficient mice are significantly larger than wild type animals and show widespread increase in skeletal muscle mass. Initial phenotypic characterization of Mst-deficient mice revealed suppression of body fat accumulation, an unexpected finding that suggested a possible role of Mst in the control of energy balance beyond its effect on skeletal muscle (McPherron and Lee, 2002). Using muscle (gastrocnemius and levator-ani) and adipose (epididymal and subcutaneous) tissues isolated from wild type and Mst-deficient male mice, Braga et al. demonstrated significant induction of BAT-related markers in both tissue types obtained from Mst-deficient mice. Protein expression levels of adipocyte specific marker adiponectin and energy sensing AMP-activated protein kinase (AMPK) was significantly upregulated in differentiating MEFs from Mst-deficient cells compared to the wild type (Braga et al., 2013). In order to clarify how Mst deficiency affects the body fat mass and energy balance, Choi et al. compared rates of oxygen consumption, body composition, and food intake in wild type, heterozygous and Mst-deficient mice and reported significantly increased energy expenditure in Mst-deficient mice (Choi et al., 2011). These studies suggested that increased fatty acid oxidation and total energy expenditure could be key reasons for reduced adiposity in Mst-deficient mice. Significantly increased muscle mass in Mst deficient mice also contributes to overall metabolism through inhibition of activin signaling, activation of AMPK/PGC-1α/Fndc5 (irisin) pathway, or by increasing Akt signaling (Guo et al., 2009; Shan et al., 2013). Subsequent studies demonstrated remarkable changes in the epididymal WAT of Mst-deficient mice compared to the wild type mice. Global gene expression analysis of these mice revealed elevated expression of genes involved in fatty acid oxidation, lipid transport, mitochondrial biogenesis, and BAT-specific transcription factors including Ucp1, Cidea, and Dio2 (Zhang et al., 2012). Histological analysis of WAT explants derived from Mst-deficient mice revealed the presence of BAT-like cells characterized by their small diameter, inclusion of multilocular lipid droplets and positive staining for UCP1. Also, these Mst-deficient mice had higher body temperature (~ 1°C) compared to the wild type mice. In the same study, the authors reported an increased abundance and activity of energy sensing enzyme AMP-activated protein kinase (AMPK) in adipose, liver, and skeletal muscle tissues of Mst-deficient mice. Subsequently several lines of evidence obtained from *in vitro* studies confirmed the inhibitory role of Mst during brown fat differentiation and regulation of energy metabolism. Brown preadipocyte cells obtained from mouse interscapular BAT, when treated with recombinant Mst protein significantly down-regulated brown adipocyte differentiation via regulation of Smad3-mediated β-catenin stabilization (Kim et al., 2012). Using primary cultures of mouse embryonic fibroblasts (MEFs) obtained from wild type and Mst-deficient embryos differentiating under specialized adipogenic conditions that allows brown adipose-like differentiation, it was demonstrated that the Mst-deficient group had significantly increased levels of select genes and proteins that improve lipid metabolism and energy expenditure (Braga et al., 2013). Treatment of differentiating Mst-deficient MEFs with recombinant Mst protein significantly inhibited the gene expression levels of key BAT markers (Ucp1, and Prdm16), general adipocyte markers (Cebp-α, and Pparγ) also expressed in BAT, metabolic regulators (Pgc-1α/β) as well as BAT inducer (Bmp7). Activation of AMPK-PGC-1α pathway in WATs of Mst-deficient mice was further confirmed by other investigators (Shan et al., 2013). Immunohistochemical staining of WAT from Mst-deficient mice showed numerous small-sized brown adipocyte-like cells filled with multilocular lipid droplets that stained with UCP1, typical characteristics of beige cells. Consistent with the browning phenotype, WAT from Mst-deficient mice also expressed significantly higher levels of beige cell specific Tmem26 and Cd137. These authors further demonstrated that loss of Mst or inhibition of Mst signaling in preadipocytes was unable to account for the browning phenotype observed in WAT of Mst-deficient mice and suggested that muscle-derived newly discovered myokine irisin (Fndc5) was responsible for the browning in a non-autonomous manner (Shan et al., 2013). Irisin was initially discovered as a PGC-1α-dependent myokine that is responsible for brown-fat-like characteristics both *in vitro* and *in vivo* and protects mice from diet-induced obesity (Boström et al., 2012). The authors further demonstrated that the effect of Mst inhibition on Pgc-1α and Fndc5 gene expression are abolished by compound C (AMPK inhibitor) suggesting that AMPK activation is required to mediate the effect of Mst on Pgc-1α and Fndc5. Activation of AMPK signaling pathway in Mst-deficient mice has also been reported previously that resulted in increased fatty acid oxidation, augmented SIRT1 signaling, and increased sensitivity to insulin (Zhang et al., 2011). The precise mechanism by which Mst inhibition may lead to the upregulation of AMPK is not clear. It is possible that Mst inhibition may regulate mTOR and FoxO1 pathways involved in muscle protein synthesis and degradation, respectively to increase the protein level of AMPK (McFarlane et al., 2006; Rodriguez et al., 2011). Most recently, Mst has been reported to inhibit AMPK in cardiomyocytes through the activation of TAK1, which is the main signal transducer of Mst besides Smads (Biesemann et al., 2014). In conclusion, these studies provide rational justification for inhibiting Mst through a novel class of Mst antagonists for treatment of obesity and related metabolic syndromes by enhancing brown adipocyte characteristics and increased energy expenditure not only in white adipocytes but also in skeletal muscle. A brief summary of TGF-β/Mst action on browning of white adipocytes and regulation of energy metabolism is shown in Figure 1.

### Follistatin regulation of brown fat and energy metabolism

**Figure 2:**
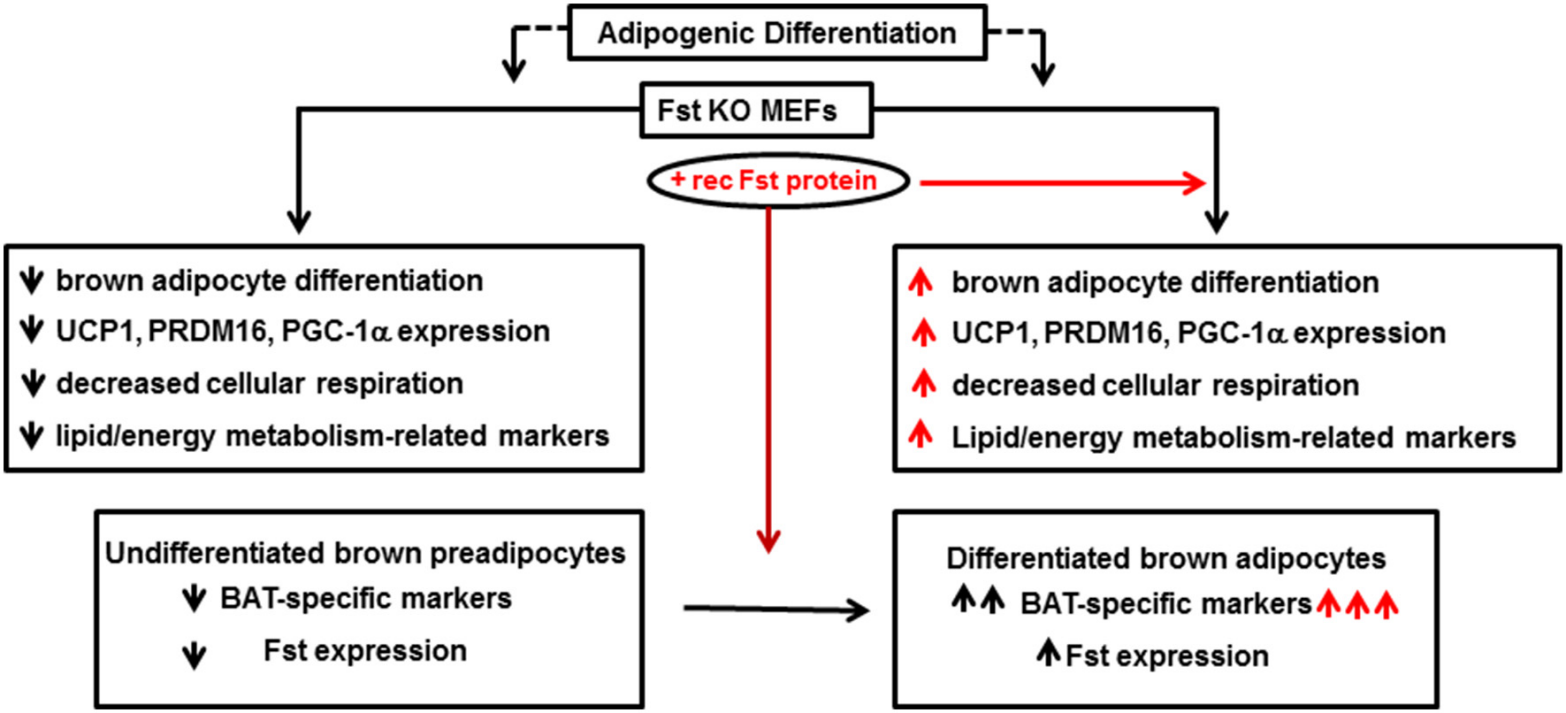
**Follistatin promotes adipocyte differentiation and energy metabolism**. Fst KO: follistatin knock out; MEF: mouse embryonic fibroblasts; UCP1: uncoupling protein 1; PRDM16: PR domain containing 16; PGC-1α: peroxisomal proliferator-activated receptor (PPAR) coactivator 1; BAT: brown adipose tissue.

Follistatin (Fst) is a secreted glycoprotein that has been recognized for many years as a high affinity binding and neutralizing protein for several members of the TGF-β superfamily including activins and Mst (Welt et al., 2002; Sidis et al., 2006). A series of genetic studies have convincingly demonstrated an essential role of Fst in skeletal muscle development (Matzuk et al., 1995; Lee et al., 2010). However, the role of Fst in other metabolic tissues is less well-known. Fst-deficient mice created by a targeted deletion of the Fst gene survived until birth but died within hours of delivery (Matzuk et al., 1995). Several defects, including reduced size of intercostal and diaphragm muscle, were observed in these mice, and these severe musculoskeletal defects were suggested as the possible cause of neonatal death for these mice. Since skeletal muscle and brown fat share Myf5^+^ as common precursor, and there is an absolute requirement of brown fat to maintain body temperature during first few hours of neonatal life, Braga et al. tested possible novel role of Fst in regulating overall thermogenic program (Braga et al., 2014). Initial gene expression analysis of a mouse tissue panel prepared from C57BL6J mice showed very high levels of Fst expression in BAT and skeletal muscle; intermediate expression in inguinal WAT and liver, and significant but lower levels in other metabolic tissues. Expression levels of Fst along with other known brown adipocyte markers were significantly induced from nearly undetectable levels after differentiation of mouse brown preadipocyte cells. Furthermore, treatment of the differentiating cells with the recombinant Fst protein further upregulated the expression of several thermogenic genes including Ucp1 and Prdm16, suggesting a possible role of Fst in the brown fat differentiation program. As direct *in vivo* analysis of lipid and energy metabolism as well as brown fat differentiation program in Fst-deficient mice was not possible because of perinatal death of Fst-deficient pups, Braga et al. isolated MEFs from wild type and Fst-deficient embryos after heterozygous breeding and allowed these cells to undergo specialized adipogenic differentiation conditions to assess possible defects in lipid and energy metabolism in Fst-deficient MEF cultures compared to the wild type (Braga et al., 2013). Surprisingly, a significant lack of stimulation of key markers for lipid and energy metabolism and mitochondrial biogenesis including Adn, Pgc1a, Bmp7, Cidea, Cyt C, Thrsp, Hp, Acsl1, Pparg, and Cd36 amongst others in Fst-deficient MEFs was observed in comparison to the wild type MEFs. Treatment of these differentiating Fst-deficient MEF cultures with recombinant Fst rescued the expression levels of these thermogenic genes and proteins. Interestingly, using a similar MEF-based approach Braga et al. also demonstrated reciprocal effects on Bmp7, Pgc1a, and Cidea gene expression in Mst-deficiency cultures compared to the wild type (Braga et al., 2013), suggesting possible cross-talk between Mst and Fst pathways during brown fat differentiation. Since PGC-1α is known to drive brown-fat-like development of white fat and thermogenesis (Boström et al., 2012), its reciprocal regulation by Fst and Mst is quite logical. BMP7, a key regulator of brown adipogenesis and energy expenditure (Tseng et al., 2008) is known to be induced by Fst (Amthor et al., 2002), but antagonized by Mst (Rebbapragada et al., 2003). Fst-deficient MEFs showed decreased oxygen consumption rate (OCR) compared to the wild type; but recombinant Fst rescued this respiration impairment indicating the crucial role of Fst during maintenance of cellular respiration. These findings therefore, suggest that Fst is a novel modulator of brown fat differentiation and energy metabolism, and its loss-of-function results in severe metabolic defects that may be essential for survival. Follistatin like-3 (FSTL3), another major Mst binding protein is reported to regulate fat mass and glucose homeostasis, it is not known whether it also regulates BAT mass and activity (Brown et al., 2011). FSTL3 knockout mice developed distinct phenotypes including increased pancreatic islet number and size, decreased visceral fat mass, and increased insulin sensitivity, suggesting that the role of Fsts may be more complicated (Mukherjee et al., 2007). The molecular mechanisms responsible for Fst-induced brown adipocyte characteristics have not been investigated; however, it is possible that Fst may antagonize components of the Mst/TGF-β signaling pathways during the process. It will be interesting to explore whether Fst antagonizes Mst/Smad3 signaling during adipocyte differentiation and browning of white adipocytes. Myf5-expressing precursor cells are found in skeletal muscle as well as in BAT. It is possible that Fst may induce classical BAT mass and its activity by directly targeting Myf5-expressing cells. On the other hand, Fst may also target pp38 MAPK/Cox-2 pathways in Myf5-negative cells to promote browning in WAT. Most recently, irisin, a PGC-1α and exercise inducible myokine has been reported to stimulate browning of WAT through p38 MAP Kinase and ERK signaling (Zhang et al., 2014). Similar to irisin, Fst levels also increase after exercise (Hansen et al., 2011). Furthermore, recombinant Fst or anti-Mst antibody treatment of mouse muscle cells increases irisin encoded Fndc5 gene (Shan et al., 2013). It is, therefore, possible that Fst may engage irisin/Mst dependent signaling to promote browning. Detailed *in vivo* studies using Fst-transgenic mice expressing Fst under the control of both white and brown adipose-specific promoters on their metabolic parameters including whole body energy expenditure, glucose tolerance, and insulin sensitivity would provide valuable information regarding potential adipocyte-specific effects of Fst. Delineating the specific tissue targets of Fst responsible for its regulation of overall energy homeostasis would provide a novel therapeutic approach for the treatment of obesity and related metabolic syndromes. Initial gene therapy preclinical studies performed on non-human primates demonstrate that sustained Fst expression caused no aberrations in the structures or functions of a variety of organs, suggesting that Fst use in clinical settings may be safe (Kota et al., 2009). A brief summary of Fst action in adipocyte differentiation and energy metabolism is shown in Figure 2.

## Summary

Several *in vitro* and *in vivo* data accumulated from various laboratories in recent years support the concept that inhibition of TGF-β superfamily/Mst either through pharmacological modulation or genetic inactivation increases brown adipose characteristics, enhances energy expenditure, and provides metabolic benefits. Therefore, inhibition of TGF-β/Mst through a novel class of antagonists could provide rationale justification not only for promoting muscle mass as expected, but also for the treatment of obesity and related metabolic syndromes. Because Fst is known to antagonize Mst and inhibit overall TGF-β signaling, investigating the potential effects of Fst on brown adipocyte characteristics is important. Current findings by Braga et al. present supporting evidence that Fst enhances the acquisition of brown adipocyte characteristics *in vitro* and has therapeutic potential for the treatment of obesity and related metabolic syndromes either alone or in combination with other drugs.

### Conflict of interest statement

Patent application (RS) entitled “Composition and methods for treating or preventing metabolic syndrome disorders” is in process. Authors declare no other potential conflict of interest.
